# Fracture Surface Behavior of 34CrNiMo6 High-Strength Steel Bars with Blind Holes under Bending-Torsion Fatigue

**DOI:** 10.3390/ma15010080

**Published:** 2021-12-23

**Authors:** Wojciech Macek, Ricardo Branco, José Domingos Costa, Jarosław Trembacz

**Affiliations:** 1 Advanced Materials Center, Gdańsk University of Technology, ul. Narutowicza 11/12, 80-233 Gdańsk, Poland; 2 Faculty of Mechanical Engineering and Ship Technology, Gdańsk University of Technology, ul. Narutowicza 11/12, 80-233 Gdańsk, Poland; 3 CEMMPRE, Department of Mechanical Engineering, University of Coimbra, 3030-788 Coimbra, Portugal; ricardo.branco@dem.uc.pt (R.B.); jose.domingos@dem.uc.pt (J.D.C.); 4 Faculty of Production Engineering and Logistics, Opole University of Technology, Prószkowska 76, 45-758 Opole, Poland; j.trembacz@po.edu.pl

**Keywords:** multiaxial fatigue, bending–torsion, high-strength steel, interacting stress concentration, multi-crack initiation, 3D fractography, surface metrology

## Abstract

The present study evaluates the fracture surface response of fatigued 34CrNiMo6 steel bars with transverse blind holes subjected to bending with torsion loading. The analysis of the geometric product specification was performed by means of height parameters *Sx*, functional volume parameters *Vx*, and fractal dimension *Df*. Surface topography measurements were carried out using an optical profilometer with focus variation technology. The experimental results show that the doubling the bending to torsion moment ratio *B/T* from *B/T* = 1 to *B/T* = 2, maintaining the same normal stress amplitude, greatly reduces both *Sa*, *Vv* as well as the fractal dimension *Df* of the analyzed specimen fractures by 32.1%, 29.8%, and 16.0%, respectively. However, as expected, a two-fold increase in the *B/T* ratio, maintaining the same normal stress amplitude, resulted in a larger number of cycles to fatigue crack initiation, Ni, which can be explained by the lower shear stress level. These experiments prove that parameters *Sx*, *Vx*, *Df* are smaller for larger *Ni* values, which is an important finding. In addition, it was found a high consistency of surface topography measurements for the two sides of the broken specimens. The proposed methodology is both reliable and applicable for other engineering applications involving different geometries and loading conditions.

## 1. Introduction

Efficient design and safe service operation of mechanical components subjected to cyclic loading is a constant goal of modern industry. Therefore, engineering materials with different geometries and shapes are increasingly studied under complex fatigue conditions and synergistic effects [1,2,3,4]. Viespoli et al. [4] studied the failure mechanisms of severe geometric discontinuities in terms of creep and fatigue interaction. They showed that the plastic behavior of the cable resulted in a minimal notch sensitivity for cracks starting from different positions associated with lower geometrical stress concentration factors. Martínez et al. [5] also have attempted to estimate the fatigue life in wires with blind micro holes. Their assessment of the geometric discontinuities in AA 6201-T81 wires showed good agreement with calculations based on Theory of Critical Distances. Loading generation also needs to be as close as possible to the real service conditions, which requires the development of specific fatigue machines either for uniaxial or multiaxial loading [6,7,8,9]. Multiaxial loading, and especially bending–torsion, is an important and interesting case, albeit quite complicated. Giannakis and Savaidis [10] took up stressed automotive antiroll bars and implemented an innovative procedure to calculate the fatigue life. Parallel to the tests, new calculation models have been developed for the determination of fatigue life, especially for loading combinations, such as bending–torsion [11,12,13]. Furthermore, a huge effort has been put on the development of advanced numerical models, particularly those based on the finite element method, to address multiaxial fatigue problems [14,15,16,17,18]. For practical applications such as cables and wire ropes [19], implantable medical leads [20], or containership structures [21]. All these methods are, in general, supplemented by material tests at various scales [22,23,24,25,26]. The morphology of the material can be examined in the depth of the material [27,28,29,30,31] and on its surface [32,33,34,35]. In some works, scientists have tried to combine all fractographic methods and link them to fracture mechanisms or fatigue life, especially using surface roughness parameters evaluated from the fracture surfaces [36,37,38]. Examples of quantitative fractography cases are presented in the works by Goldsmith et al. [39] and by Kobayashi et al. [40]. However, as far as the authors know, there are no studies in the literature connecting multiaxial loadings and surface fracture parameters in round bars with blind holes subjected to multiaxial loading. 

Thus, this paper attempts to link the fracture surface topography parameters, including fractal dimension *Df*, with the multiaxial fatigue loading, particularly bending–torsion loading, in circular cross-section geometries containing transversal blind holes. Surface analysis was taken out on the whole fracture surface area, without partition into the three fatigue stages, i.e., initiation, propagation, and final rupture. More specifically, the paper aims to investigate the effect of the bending moment to torsion moment ratio on fatigue crack initiation of round bars with transversal blind holes; and on fracture surface topography. It is also focused on the identification and optimization of surface topography parameters in the context of fatigue crack mechanisms, where fracture topography is evaluated via the entire fracture area method [41,42,43].

Following the Introduction, the paper is organized as follows: Section 2 describes the materials and methods used in this research. Section 3 gathers information on the experimental fatigue behavior and the fracture surface fractography results. Section 4 presents the main outcomes about the fracture surface analysis conducted using different fractographic parameters for both sides of the specimens. The paper ends with a summary of the most relevant findings. Finally, for the sake of clarity, two appendixes were added: Appendix A shows the 3D views for the investigated fracture surfaces, both before and after extracting the region of interest (ROI), while Appendix B plots four variants of calculating the fractal dimension with its parameters.

## 2. Materials and Methods

### 2.1. Material and Fatigue Test Procedure

The material tested in this research was the DIN 34CrNiMo6 high strength steel, a martensitic steel, whose elemental composition and mechanical properties are summarized in Table 1 and Table 2, respectively. This steel exhibits a martensitic matrix containing small amounts of ferrite and bainite. The microstructure is the result of a rapid quenching that transforms most of the austenite into martensite. The strengthening mechanisms are associated with the precipitation of a fine dispersion of alloy carbides during tempering.

**Figure 1:**
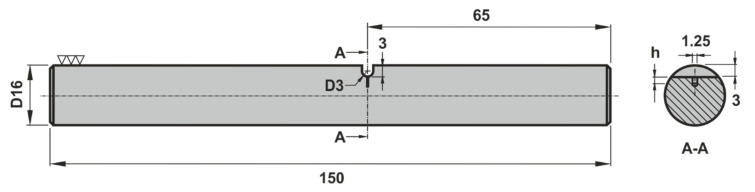
Specimen geometry used in the bending–torsion fatigue tests [44].

The bending–torsion fatigue testing program, originally performed in a previous study, has been conducted in a 100 kN DARTEC servo-hydraulic machine (Dartec Ltd., Bournemouth, UK) connected to a custom-made gripping system [44]. The specimens, whose geometry is presented in Figure 1, were prepared in a high-precision computer numerical control turning center from extruded 20 mm-diameter round bars and were tested under in-phase constant-amplitude for pulsating loading conditions (*R* = 0). This circular cross-section geometry encompasses a lateral U-shaped notch along with a 1.25 mm-diameter central hole whose depth (h) varies between 0.3 and 1.4 mm (see Table 3). 

The tests were performed in air, at room temperature, with sinusoidal waves, and cyclic frequencies in the range 3–6 Hz, using a conventional servo-hydraulic machine connected to a custom-made gripping system. More details about the experimental apparatus can be seen in the paper by Branco et al. [45] Table 3 precises, inter alia, the nominal normal stress amplitude (*σ_a_*) and the nominal normal mean stress (*σ_m_*) applied in each individual specimen during the tests. The bending moment to torsion moment ratios, *B/T*, also presented in Table 3, were equal to 1 and 2. The number of cycles to fatigue crack initiation for each case was calculated using the El-Haddad parameter (*a*_0_) which can be defined by the following equation:

(1) $$a_{0}=\frac{1}{\pi }{\left(\frac{\Delta K_{th}}{\Delta \sigma_{0}}\right)}^{2}$$

where $\Delta K_{th}$ is the range of the threshold value of the stress intensity factor and $\Delta \sigma_{0}$ is the fatigue limit stress range of the unnotched specimen. The two constants are evaluated under the same stress ratio (R = 0, in this case) of the multiaxially loaded geometry. The values obtained in this research, originally calculated in a previous study [44], are listed in Table 3.

### 2.2. Fracture Surface Measurement

The fracture surface parameters were determined using a profilometer (Alicona Imaging GmbH, Graz, Austria) using the Focus Variation Method. This non-contact measuring system uses a white light source to project light beams onto the specimen’s surface. More information about the methodology used to carry out the surface texture evaluations can be found in the paper by Macek et al. [46]. Reflected light rays appear from the measured surface and are processed via a precise sensor. In this study, the total area of the fracture surface was investigated using an objective magnification of 10×. The main measurement parameters are summarized in Table 4. To perform the scanning of the total area, the imagefield function was used. The fracture surface was divided into 19 rows and 13 columns, and then the individual images were stitched together to map the entire fracture region. 

Surface fractography studies were carried out on the entire fracture area using height parameters *Sx* and functional parameters (volume) *Vx* defined according to ISO 25178 [47], as well as the fractal dimension *Df*. This standard, whose main title is “Geometrical product specifications (GPS)—Surface texture: Areal”, introduces the terminology and the main definitions associated with surface texture, and describes the main parameter used in the evaluation of surface texture. Regarding the Sx parameters, as defined in Table 5, *Sq* and *Sa* are the root-mean square height and the arithmetical mean height of the surface, respectively; *Sz* is the maximum height of the surface, that is, the height between the highest peak and the deepest valley; the maximum peak height, *Sp*, is calculated as the difference for height between the highest peak and the deepest valley, *Sz*, and the maximum pit height, *Sv*. Skewness *Ssk* is a measure of the symmetry of the height distribution and can thus be used to point at superiority of peaks on the surface for *Ssk* > 0 whether valley for *Ssk* < 0. In pursuance of Krolczyk et al. [48], kurtosis *Sku* indicates appearance on the surface of excessively high peaks or deep valleys for *Sku* > 3, or their absence on the surface for *Sku* < 3. The functional parameters included: *Vm*, *Vv*, *Vmp*, *Vmc*, *Vvc*, and *Vvv* [47,49]. Table 5 defines the selected parameters according to the ISO 25178 standard. 

**Figure 2:**
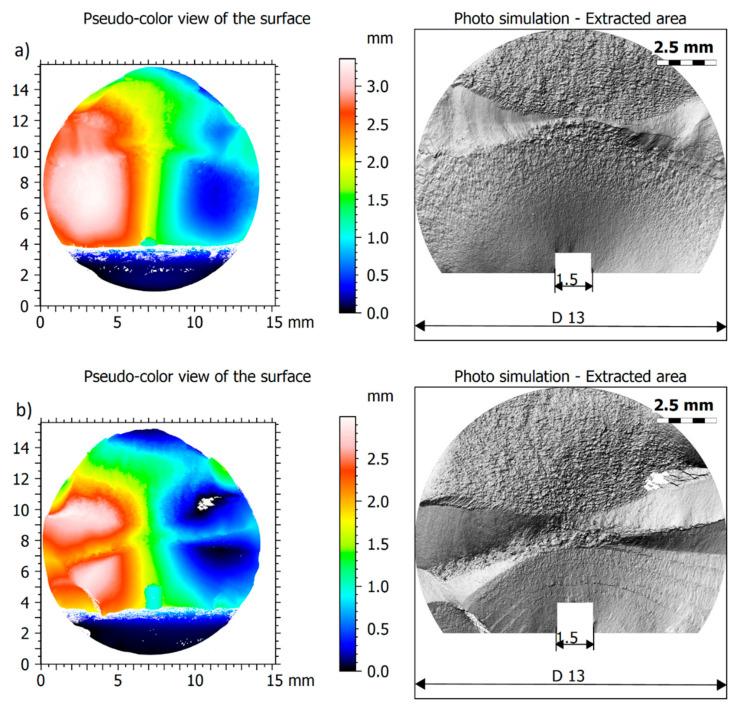

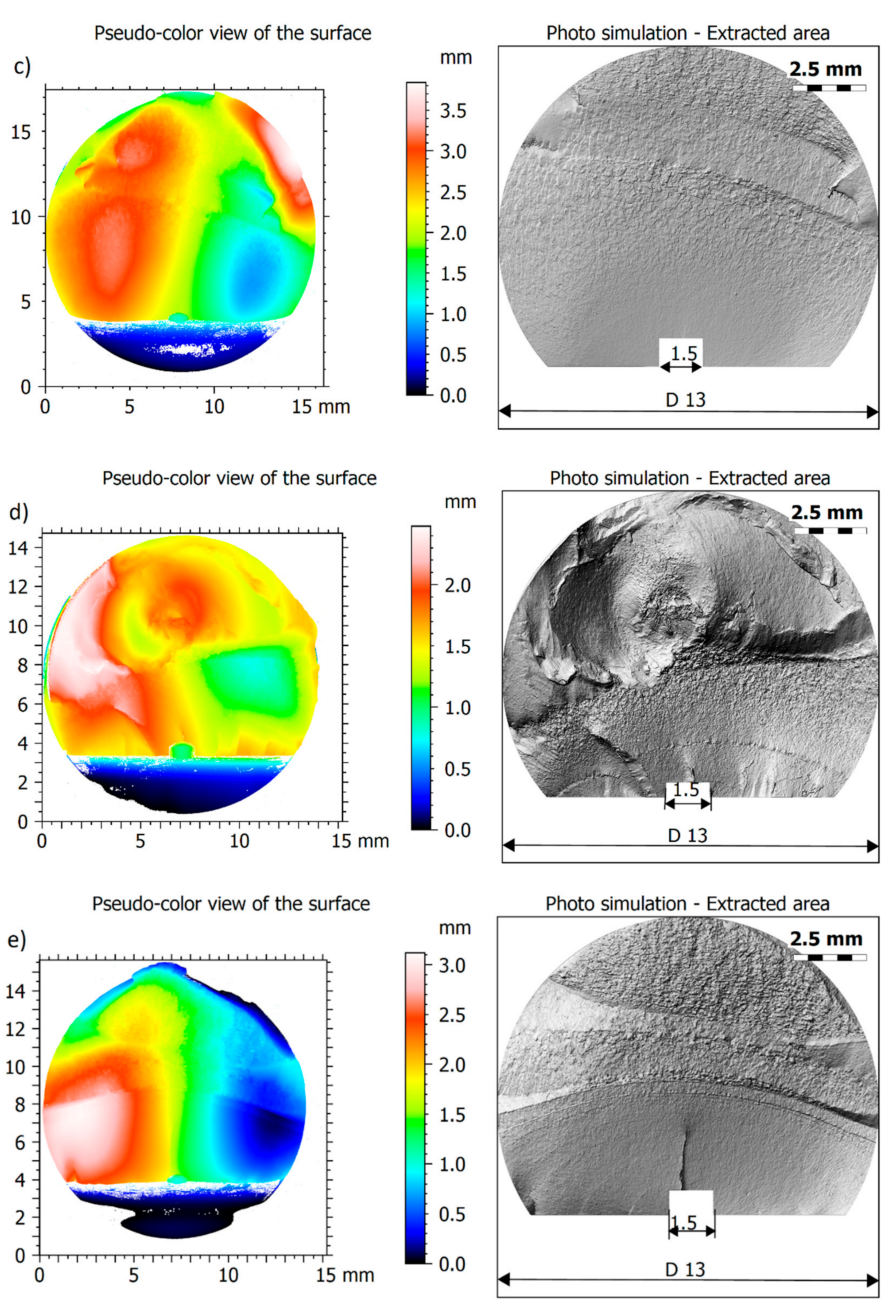
Original and extracted fracture surfaces: (**a**) BT1-2; (**b**) BT1-3; (**c**) BT2-1; (**d**) BT2-2; (**e**) BT2-3.

The fractal dimension *Df* was calculated using the enclosing boxes method (EBM) from the extracted final fracture surface areas (see Figure 2) with four variants of calculations, i.e., EBM and EBM in real units for two resolutions: *coarse* and *fine*. The EBM divides the profile into smaller sections with a width *ε* and calculates the field *Aε* of all fields covering the entire profile [50,51]. This is an iterative procedure in which the width of the field is changed to plot, ln(*Aε*)/ln(*ε*). The EBM in real units considers real Z-spacing values to calculate the enclosed area. The resolution of the graph determines the number of iterations and, therefore, the calculation time. For the *fine* resolution, 59 points in the plot were considered, while for the *course* resolution, the analysis used 16 points.

**Table 5:** Selected parameters for fatigue fracture surface description according to ISO 25178 [47,52].

Height Parameters (*Sx*), ISO 25178			
*Sq*	µm	Root-mean-square height	$Sq=\sqrt{\frac{1}{A}\iint_{A}^{}\;z^{2}\left(x,y\right)dxdy}$
*Sv*	µm	Maximum pit height	Absolute value of the height of the largest pit within the defined area
*Sz*	µm	Maximum height	Height between the highest peak and the deepest valley
*Sa*	µm	Arithmetical mean height	$Sa=\frac{1}{A}\iint_{A}\;\left|z\left(x,y\right)\right|dxdy$
*Sp*	µm	Maximum peak height	Sp = Sz - Sv
*Ssk*	-	Skewness	$Ssk=\frac{1}{Sq^{3}}\iint {\left(z\left(x,y\right)\right)}^{3}dxdy$
*Sku*	-	Kurtosis	$Sku=\frac{1}{Sq^{4}}\iint {\left(z\left(x,y\right)\right)}^{4}dxdy$
**Functional Parameters (Volume)(*Vx*), ISO 25178**			
*Vm*	mm^3^/mm^2^	Material volume	Parameters describing the characteristics of the volume of the appropriate size to the surface area of the surface being examined
*Vv*	mm^3^/mm^2^	Void volume	
*Vmc*	mm^3^/mm^2^	Core material volume	
*Vmp*	mm^3^/mm^2^	Peak material volume	
*Vvv*	mm^3^/mm^2^	Pit void volume	

Where *A* is the definition area; *z* is the surface height in position *x*, *y; x*, *y* are the lengths in perpendicular directions.

## 3. Results

Table 6, Table 7 and Table 8 summarize the main variables of the fracture surface measurements, namely *Sx* and *Vx*, and fractal dimension *Df*, respectively, for the different specimen geometries subjected to in-phase bending–torsion loading. For the *B/T* = 2 ratio, both sides of the same specimen (BT1-3a and BT1-3b) were evaluated to study the consistency of surface topography measurements based on the two fracture surfaces. 

**Figure A1:**
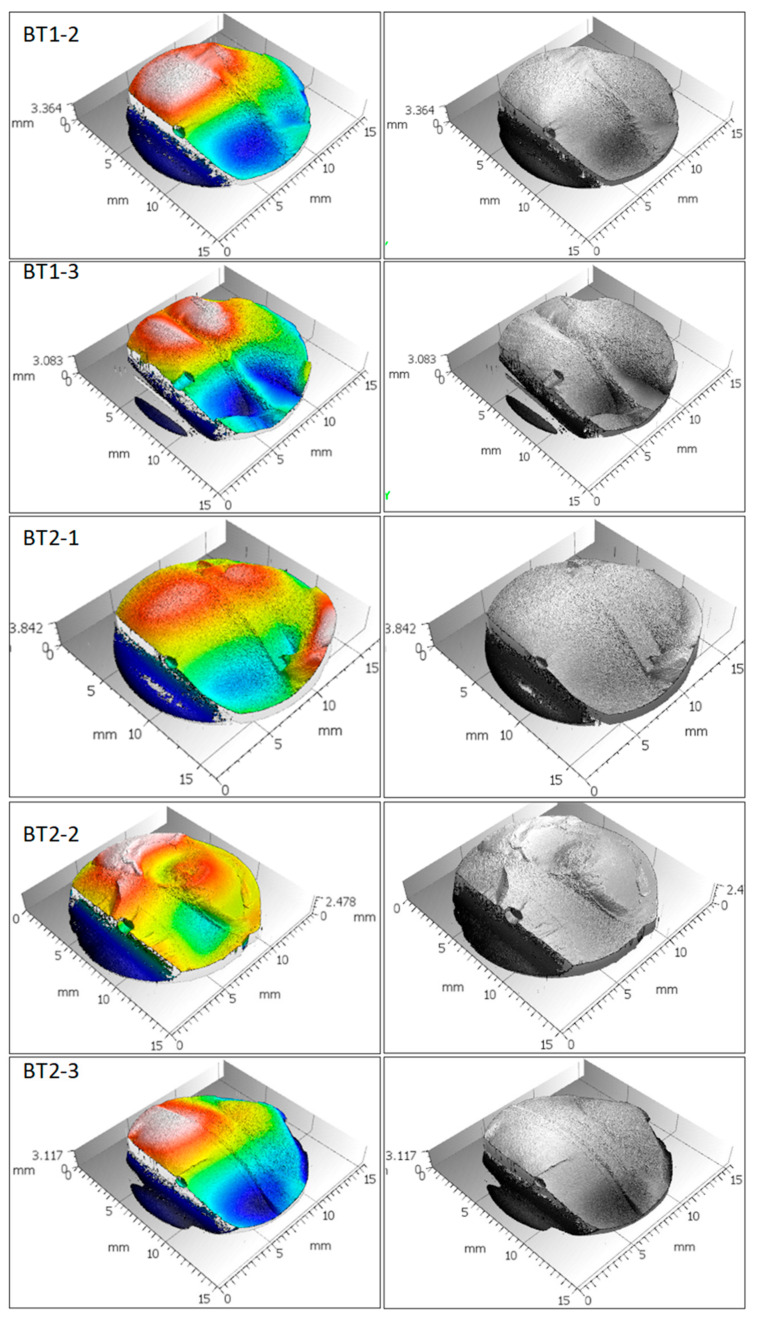
Original measured surfaces of fracture.

**Figure A2:**
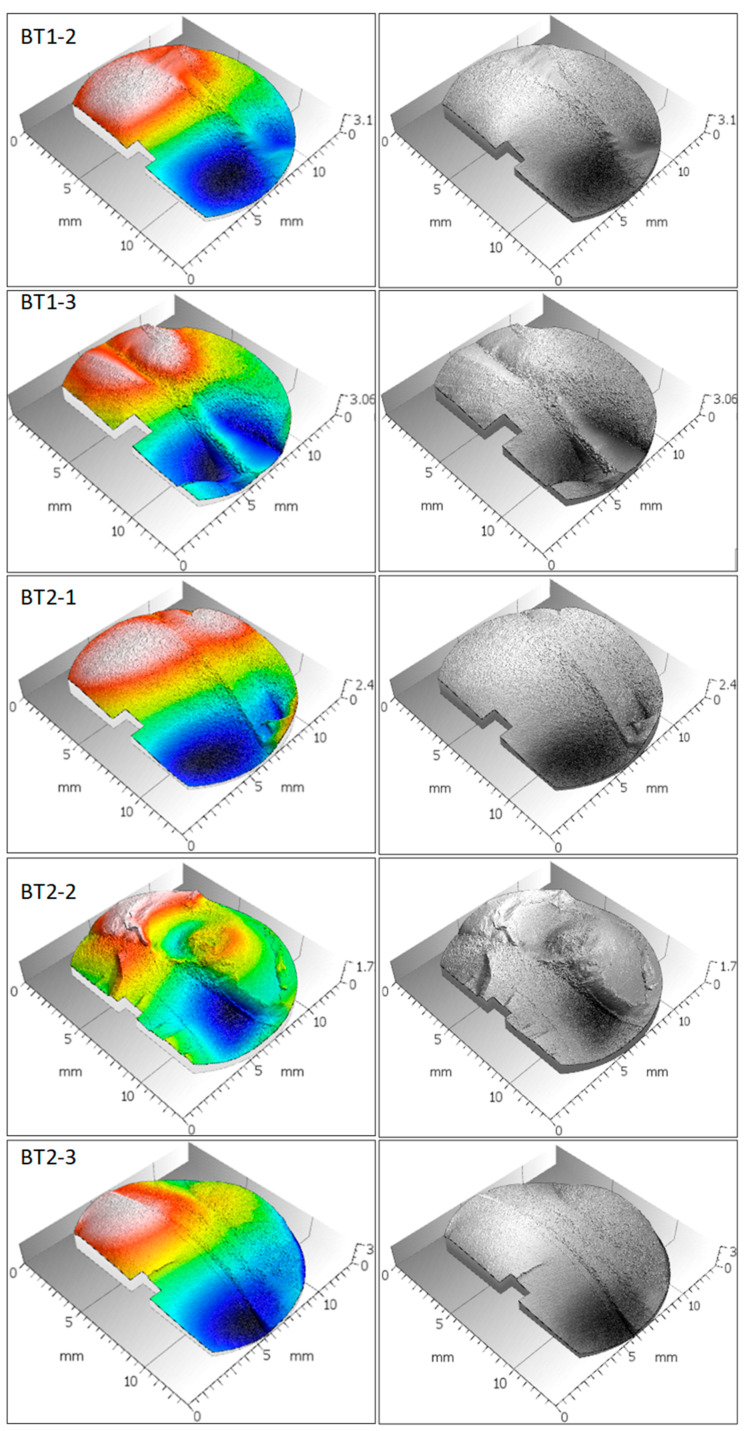
Extracted surfaces of fracture reduced to the region of interest (ROI).

The fracture surfaces of 5 selected specimens, previously subjected to fatigue loading, were measured. All fractographic parameters were calculated on the whole fracture surface. The entire surface was reduced to eliminate the final break, discontinuities and “non-sampling” areas. Original pseudo-color views of the fracture surfaces, on the left-hand side, and photo simulations of extracted fractures areas, on the right-hand side, are presented in Figure 2. All analyzed samples in their original state as well as the corresponding extracted areas are presented in Appendix A, in Figure A1 and Figure A2, respectively.

**Figure 3:**
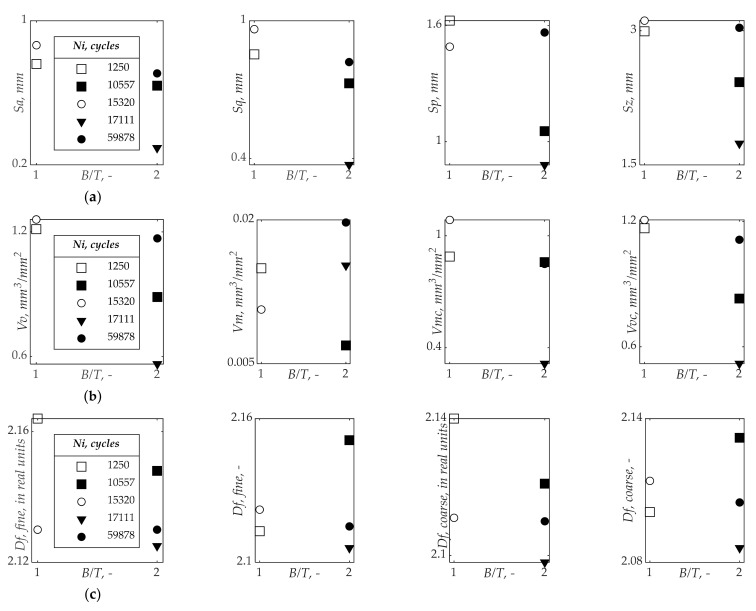
Results of fracture surface measurement: (**a**) *Sx* parameters; (**b**) *Vx* parameters; and (**c**) *Df* parameters grouped by *N_i_*.

Figure 3 presents a summary of results of the fracture surface measurements, as a scatter plot, for the different tested specimens. It is clearly seen that all values of both *Sx* and *Vx* are higher for *B/T* = 1 (*σ/τ* = 2) than for *B/T* = 2 (*σ/τ* = 4). A similar tendency can be noticed for the fractal dimension, *Df*, determined by the EBM described above. However, its values show greater differentiation and dispersion at *B/T* = 1.

## 4. Discussion

### 4.1. Cracking Mechanisms

**Figure 4:**
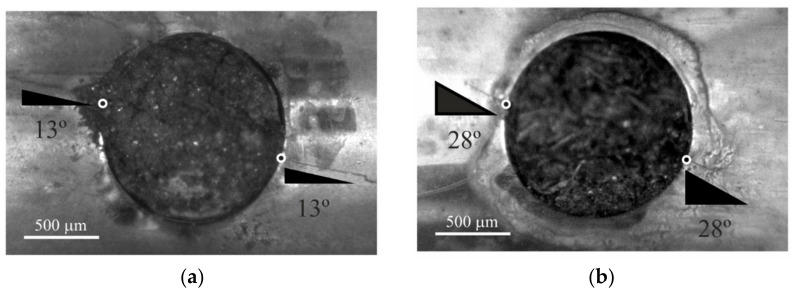
Experimental surface crack paths at the early stage of growth and crack initiation locations; (**a**) *B/T* = 2; and (**b**) *B/T* = 1 [44].

The typical locations of fatigue crack initiation as well as the fatigue crack paths at the early stage of growth at the notch surface for the two *B*/*T* ratios are exhibited in Figure 4. In this geometry, as can be seen, there is a multi-crack initiation phenomenon. Two cracks appear at the hole surface in diametrically opposite coordinates. These locations are affected by the loading scenario. For higher *B*/*T* ratios, the angle formed by the line that connects the two initiation sites (black and white dots) is lower, which is explained by the fact that the crack is closer to Mode-I (see Figure 4a). In the absence of shear stresses, this angle should be 0°. On the other hand, as the *B*/*T* ratio decreases, i.e., the shear stress level increases, this angle rises, which is associated with the higher degree of mixed-mode loading of these cases (see Figure 4b). 

Another important difference is concerned with the crack paths at the early stage of growth. As can be seen in Figure 4, these angles are similar for both sides of the hole, respectively equal to 13° for *B/T* = 2 and 28° for *B*/*T* = 1. It is also clear that this angle is affected by the loading scenario. The increase of the *B/T* ratio leads to smaller angles which can be justified by the different shear stress levels of the two loading cases. As referred to above, the crack front subjected to higher *B/T* ratios is closer to Mode-I, while the other is subjected to a higher degree of mixed-mode loading [53]. 

**Figure 5:**
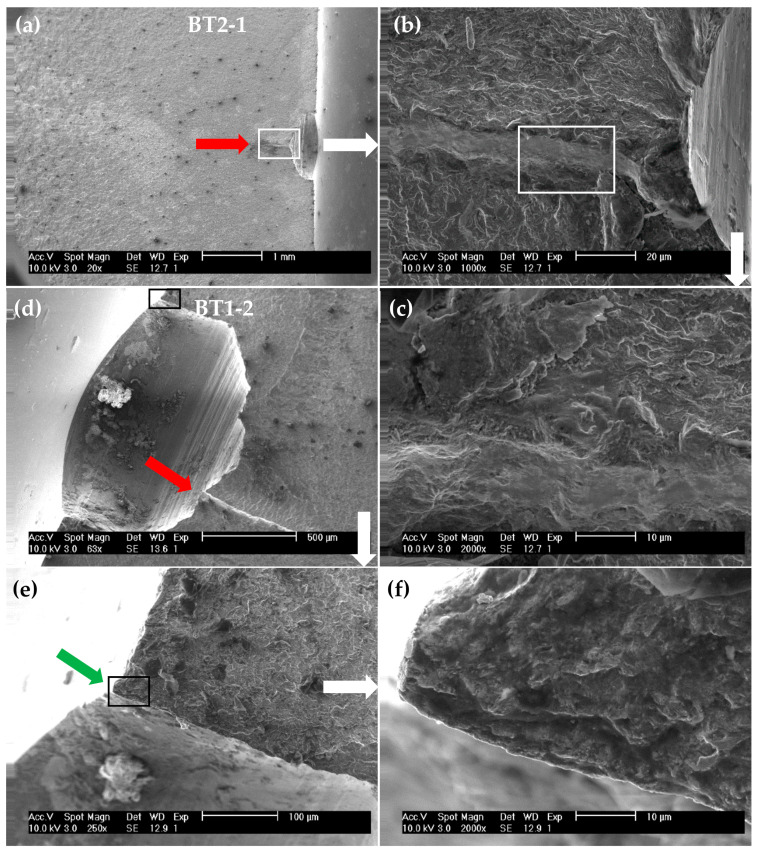
SEM micrographs of the BT2-1 (**a**–**c**) and the BT1-2 (**d**–**f**) example.

Figure 5 shows the typical aspect of fracture surfaces observed by scanning electron microscopy (Zeiss, Jena, Germany) of the multiaxial fatigue tests for *B*/*T* = 2 (Figure 5a–c) and *B*/*T* = 1 (Figure 5d–f). Overall, as can be expected, we can see the main micro-mechanisms associated with cyclic loading, namely traces of plastic deformation and secondary cracks. The multi-crack initiation phenomenon can be also inferred from the images. In general, when two cracks coalesce, it is visible a fatigue step is caused by the junction of two different planes of propagation. Representative examples of fatigue steps are exhibited in Figure 5a,d (see the red arrows). Particularly in the former case, coalescence of both cracks occurred at the middle point of the hole. In the other case, this junction of both propagation planes is slightly deviated to one side of the hole. The traces of plastic deformation caused by cyclic loading are clearly visible in Figure 5b,c which show a magnification of the surface region near the hole boundary in the area where both cracks coalesced. Figure 5e,f shows the fracture surface close to the initiation site (i.e., at the hole boundary). It is possible to see the radial convergence of the fatigue marks to the vertex (see the green arrow) caused by the cyclic loading. 

**Figure 6:**
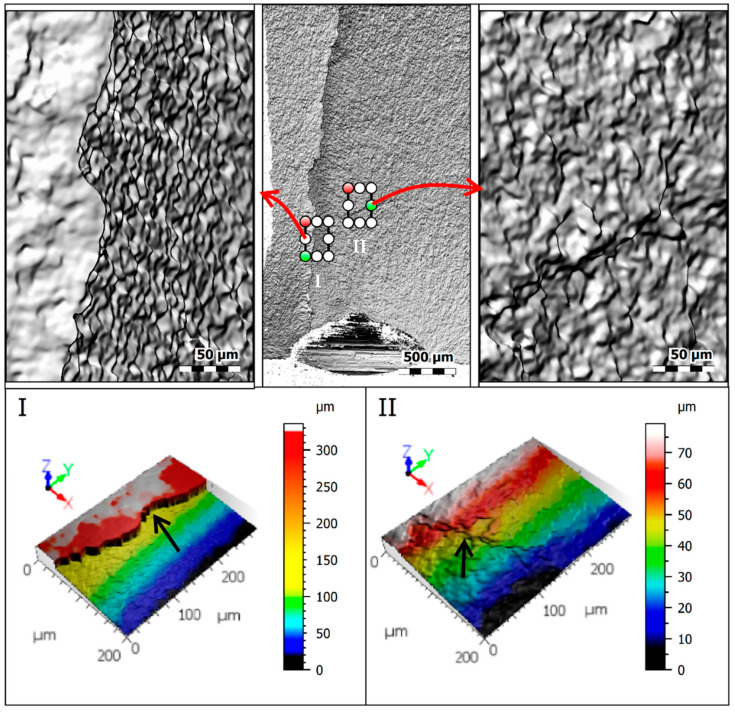
Fracture surface of the BT2-3 specimen near the fatigue step (Region I represents a fatigue step, and Region II represents the propagation region).

The region where the crack coalesced for the case BT2-1 (see Figure 5a–c) is analyzed, in more detail, in Figure 6 using profilometer scans. Zone I is obtained on the fatigue step which resulted from the junction of both cracks initiated at the hole surface in diametrically positions of the circular boundary (see Figure 4); while Zone II was taken in a region close to the fatigue step but in a region not affected by the junction of both cracks. As can be seen, the differences in the z-axis coordinates represented by the pseudo-color views are significant. In the former case, the maximum values are more than three times greater than the latter case.

### 4.2. Fractured Specimen Both Sides Comparison

**Figure 7:**
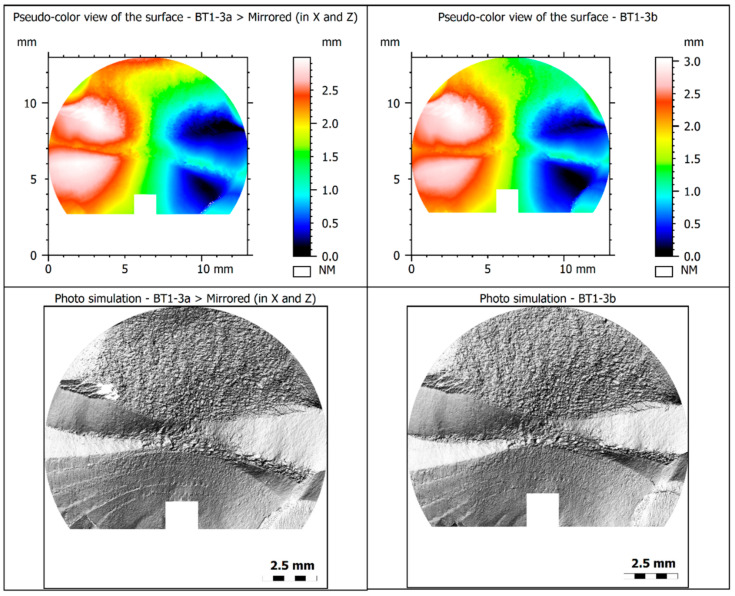
Both sides of fracture surfaces of the specimen (BT1-3). The figure on the left-hand side has been mirrored with respect to the x-axis.

An important issue in fractographic analysis of fracture surfaces caused by fatigue loading is the coherency degree of the measurements carried from both sides of the same specimen. In order to check whether the fracture surfaces obtained for the tested cases can be analyzed using any of the two sides of the same specimen, a comparison for a specific case is presented in Figure 7, which visually compares the fracture surfaces of the two sides of the BT1-3 specimen. The validation was made by mirroring the BT1-3a side with respect to the *x-*axis by inverting the *x*-coordinates and the heights of the *Z-*axis. Overall, both figures are similar, either in the original figures or in the pseudo-color representation, which suggests that the fractographic parameters are likely to be quite similar. The surfaces prepared in this way, together with the measurement results, could also be used for further comparative analyses. 

**Figure 8:**
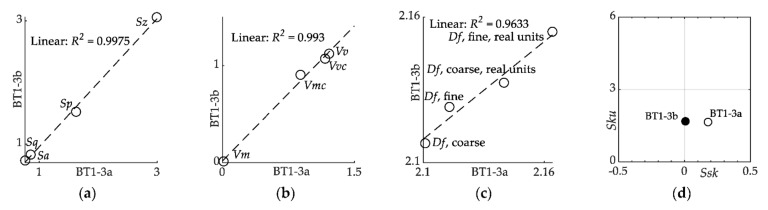
Relationship between surface parameters of both sides of the BT1-3 specimen: (**a**) height *Sx*; (**b**) functional (volume) *Vx* parameters; (**c**) fractal dimension *Df* parameters; and (**d**) kurtosis *Sku* and skewness *Ssk* map.

Figure 8a,b present a comparison carried out using the *Sx* and *Vx* parameters for both sides of specimen, respectively, excluding *Sku* and *Ssk*, whose map is shown in Figure 8d. Both the *Sx* and *Vx* parameters showed very high compliance for both sides of the specimen, with coefficients of determination close to 1, which confirms the independence of these parameters relatively of the mounting of the specimen on the gripping system. The same is true for the fractal dimension *Df* (see Figure 8c), whose coefficient of determination *R^2^* is equal to 0.9633. 

The *Ssk* parameter provides information about the asymmetry of the surface. The *Ssk* parameter value indicates the predominance of peaks on the surface for *Ssk* > 0. The *Sku* parameter demonstrates absence of inordinately high peaks or deep valleys for *Sku* < 3. As can be seen in Figure 8d, there are no dependencies on the specimen side for the *Ssk* and *Sku* parameters.

### 4.3. Effect of B/T Ratio on the Fracture Surface Parameters

**Figure 9:**
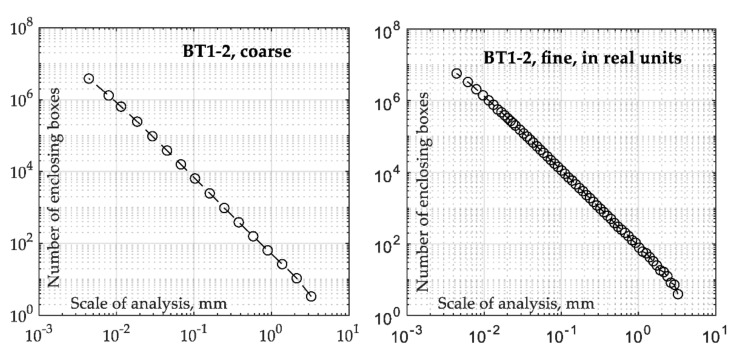
Fractal dimension *Df* data obtained using individual parameters for the BT1-2 specimen.

In order to better understand the effect of *B/T* ratio on fracture surface parameters, a detailed analysis based on the *Sa* and *Vv* parameters was performed. These parameters turned out to be the most fitted, which was also confirmed in the papers [41,43]. Moreover, for the *Df* parameters, the one determined using the EBM in real units with fine resolution was selected as the most accurate. Selected cases of the EBM estimations for the BT1-2 specimen are shown in Figure 9 for the sake of clarity. In addition, all fractal dimension *Df* plots and parameters for the four calculation conditions considered in this study are presented in Appendix B. To estimate the fractal dimension *Df* a line is fitted using the least-squares method. The absolute value of the slope of the fitted line is the estimation of the fractal dimension *Df*. The densification of the measurement points increases the slope of the curve angle, which is reflected in a slightly higher value of the fractal dimension *Df*.

**Figure 10:**
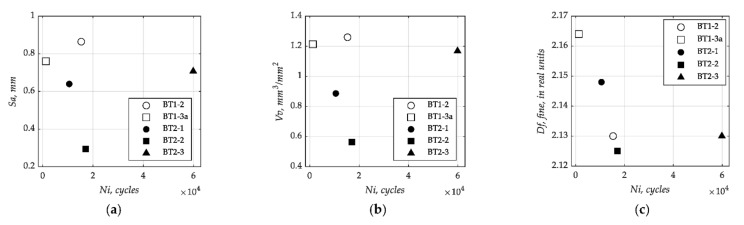
(**a**) Arithmetical mean height *Sa* parameter against the number of cycles to fatigue crack initiation *N_i_*; (**b**) void volume *Vv* parameter against the number of cycles to fatigue crack initiation *N_i_*; and (**c**) fractal dimension *Df* against the number of cycles to fatigue crack initiation *N_i_*.

Figure 10 plots the dependence of selected surface parameters (*Sa*, *Vv*, *Df*) on the number of cycles to fatigue crack initiation, *N_i_*. Samples marked with blank markers are for the cases of *B/T* = 1 while the filled markers correspond to the cases of *B/T* = 2. The analysis of results shows that the former cases have higher roughness values and earlier fatigue crack initiation lives for the same nominal normal stress level. These dependencies are the similar for both selected height parameters *Sx* or *Sa* (see Figure 10a) and for both functional parameters *Vx* or *Vv* (see Figure 10b). Regarding the fractal dimension *Df*, which is represented in Figure 10c, the values for the *B/T* = 2 show a similar trend than those presented in the Figure 10a,b. On the contrary, for the cases *B/T* = 1, the values are more distant and show an opposite trend, i.e., the values of the BT1-3a case is higher than the value of the BT1-2 case. 

**Figure 11:**
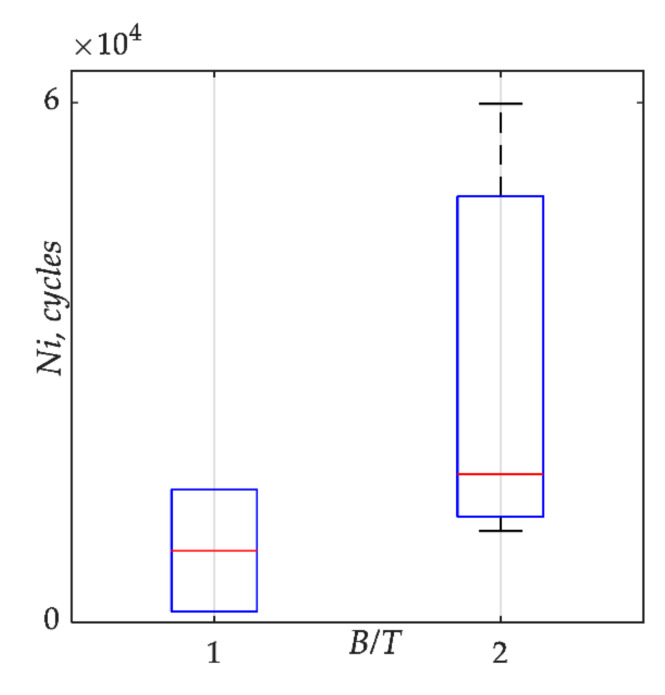
Boxplot for number of cycles to crack initiation *Ni* values including bending/torsion moment ratio *B/T*.

Figure 11 present a boxplot where on both boxes, the central mark indicates the median, and the bottom and top edges of the box indicate the 25th and 75th percentiles, respectively. The whiskers extend to the most extreme data points. Moreover, the increase of the *B/T* ratio, which causes a reduction of the shear stress level, leads to a larger number of cycles to fatigue crack initiation *Ni.* This shows that the probability of a faster fatigue crack initiation increases for higher *B/T* ratios. 

**Figure 12:**
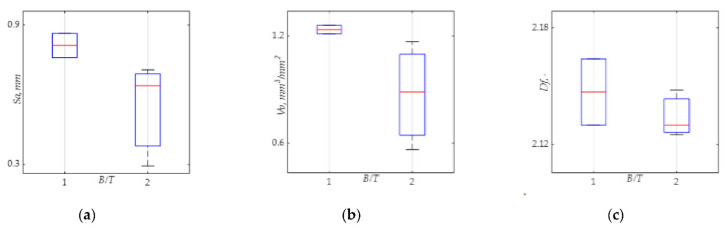
Boxplot for: (**a**) arithmetical mean height *Sa*, and (**b**) void volume *Vv* parameters, (**c**) fractal dimension *Df*.

Figure 12 present the three boxplots, showing the average values of the surface parameters *Sa*, *Vv* and *Df* for the *B/T* ratios studied in this research. Overall, as can be distinguished in the figure, regardless of the parameter used in the analysis, we can obtain the same relationships. i.e., the smallest average values of *Sa*, *Vv* and *Df* occurred for *B/T* = 2. The values of the *Sa*, *Vv* and *Df* parameters calculated from the fracture surfaces of the tested specimens are reduced 32.1%, 29.8%, and 16.0%, respectively.

This methodology, connecting the applied nominal loading with the fracture mechanics based on topographic parameters, provides important clues to improve the materials performance as well as to mitigate the fatigue damage mechanisms [32,41,42,43,46]. It can be used, for instance, in the field of forensic engineering to trace back to the origin of structural failures and correlate them with the applied loads, establishing the dependence between the loading scenario and the characteristic features of their surfaces.

## 5. Conclusions

The effect of the bending moment to torsion moment ratio (*B/T*) on fracture surface parameters in notched round bars made of high-strength steel was studied. Two different values of *B/T* (2 and 1) were considered in the multiaxial fatigue testing program. After the fatigue tests, a quantitative analysis of the entire fracture surface of broken specimens was performed using height parameters *Sx*, functional volume parameters *Vx*, and fractal dimension *Df*. The following conclusions can be drawn as follows:

− The approach of analyzing the entire surface of the fracture is a valid concept when trying to estimate the causes of the destruction of high-strength steels subjected to bending–torsion loading;
− Height (*Sx*), functional (*Vx*) and fractal dimension *Df* fracture surface texture parameters determined in the entire area of the fracture surface showed dependence on bending moment to torsion moment ratio;
− The bending moment to torsion moment ratio has a strong influence on the crack initiation sites, crack paths in the early stage of growth, and the number of cycles to fatigue crack initiation lives.
− The number of cycles to fatigue crack initiation, which is closed related to the loading scenario and stress level, significantly affects the height (*Sx*), functional (*Vx*) and fractal dimension *Df* fracture surface texture parameters;
− The comparison of the surface topography measurements obtained for the two fracture surfaces of the same specimen demonstrated an independence of these parameters relatively to the specimen side selected in the analysis;
− Resolution used in the EBM has a significant impact on the results of the calculated fractal dimension *Df*. The most accurate values in this study were those based determined in real units with fine resolution;
− Regardless of the surface texture parameters used, it was found that the smallest average values of *Sa*, *Vv* and Df occurred for the higher bending moment to torsion moment ratio.

## Figures and Tables

**Table 1:** Nominal chemical composition (wt.%) of 34CrNiMo6 high-strength steel [44].

C	Si	Mn	Cr	Mo	Ni
0.34	≤0.40	0.65	1.50	0.22	1.5

**Table 2:** Mechanical properties of the 34CrNiMo6 high-strength steel [44].

Mechanical Property	Value
Yield strength, *σ_YS_* (MPa)	967
Tensile strength, *σ_UTS_* (MPa)	1035
Young’s modulus, *E* (GPa)	209.8
Poisson’s ratio, *ν*	0.296

**Table 3:** Summary of the multiaxial fatigue test program [44].

Specimen	*B/T*	*D* (mm)	*h* (mm)	$\sigma_{a}\;(\mathrm{MPa})$	$\sigma_{m}\;(\mathrm{MPa})$	*N_i_* (Cycles)
*B/T* = 2 (σ_a_/τ_a_ = 4, σ_m_/τ_m_ = 4)						
BT2-1	2	16	0.3	224	239	10,557
BT2-2	2	14	0.6	179	194	17,111
BT2-3	2	14	0.3	179	194	59,878
*B/T* = 1 (*σ_a_*/*τ_a_* = 2, *σ_m_*/*τ_m_* = 2)						
BT1-2	1	14	0.5	179	194	15,320
BT1-3	1	14	1.4	298	313	1250

**Table 4:** Alicona G4 measurement device main parameters.

Parameter	Value
Magnification	10×
Vertical resolution	57.3 nm
Lateral resolution	3.91 µm
Number of images	19 rows × 13 columns
Exposure time	168.5 µs
Contrast	0.46

**Table 6:** Summary of the *Sx* results.

Specimen	*Sq*	*Ssk*	*Sku*	*Sp*	*Sv*	*Sz*	*Sa*
*B/T* = 2 (*σ_a_/τ_a_* = 4, *σ_m_/τ_m_* = 4)							
BT2-1	0.727513	−0.30411	1.738607	1.053918	1.371258	2.425176	0.639445
BT2-2	0.372512	0.191031	2.689261	0.879802	0.859294	1.739096	0.293833
BT2-3	0.819989	0.106609	1.858366	1.563237	1.469907	3.033144	0.707707
*B/T* = 1 (*σ_a_/τ_a_* = 2, *σ_m_/τ_m_* = 2)							
BT1-2	0.963171	−0.05294	1.552058	1.489988	1.622898	3.112886	0.86415
BT1-3a *	0.853733	0.177983	1.650938	1.624546	1.36942	2.993966	0.760138
BT1-3b *	0.831473	0.006956	1.681689	1.526147	1.533535	3.059682	0.735659

* 3a and 3b are from the same test (both sides of specimen).

**Table 7:** Summary of the *Vx* results.

Specimen	*Vm*	*Vv*	*Vmp*	*Vmc*	*Vvc*	*Vvv*
*B/T* = 2 (*σ_a_/τ_a_* = 4, *σ_m_/τ_m_* = 4)						
BT2-1	0.025494	0.429055	0.025494	0.31325	0.392693	0.036362
BT2-2	0.010651	1.259677	0.010651	1.084705	1.207675	0.052001
BT2-3	0.014953	1.213044	0.014953	0.888243	1.167677	0.045367
*B/T* = 1 (*σ_a_/τ_a_* = 2, *σ_m_/τ_m_* = 2)						
BT1-2	0.015251	0.563484	0.015251	0.314601	0.518959	0.044525
BT1-3a	0.019732	1.168728	0.019732	0.848676	1.112868	0.05586
BT1-3b	0.013873	1.123438	0.013873	0.906254	1.072114	0.051325

**Table 8:** Summary of the *Df* results, with four different calculation parameters.

Specimen	EBM, Coarse Resolution	EBM, Fine Resolution	Ebm in Real Units, Coarse Resolution	Ebm in Real Units, Fine Resolution
*B/T* = 2 (*σ_a_/τ_a_* = 4, *σ_m_/τ_m_* = 4)				
BT2-1	2.132	2.151	2.121	2.148
BT2-2	2.086	2.106	2.098	2.125
BT2-3	2.105	2.115	2.110	2.130
*B/T* = 1 (*σ_a_/τ_a_* = 2, *σ_m_/τ_m_* = 2)				
BT1-2	2.114	2.122	2.111	2.130
BT1-3a	2.101	2.113	2.140	2.164
BT1-3b	2.132	2.151	2.121	2.148

**Figure A3:**
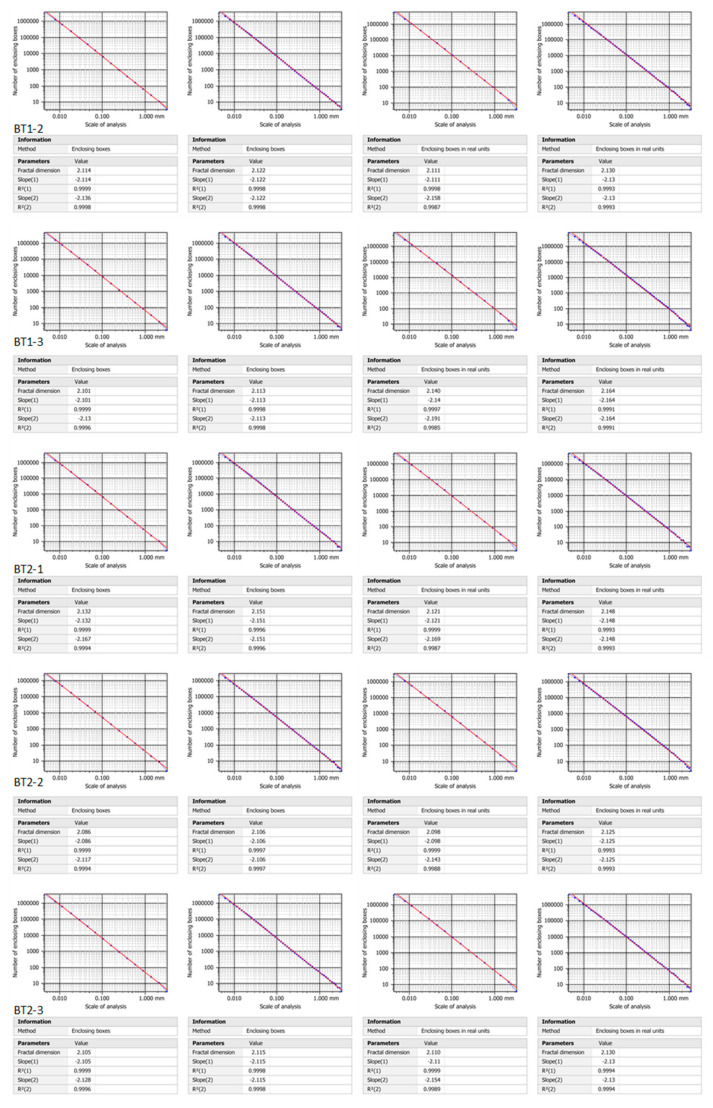
Fractal dimension *Df* plots.

## Data Availability

Not applicable.

## Nomenclature

*A*	mm^2^	area
*B/T*	-	bending moment to torsion moment ratio
*D*	mm	specimen diameter
*Df*	-	fractal dimension
*E*	GPa	Young’s modulus
*h*	mm	hole depth
*Ni*	cycles	number of cycles to crack initiation
*R*	-	stress ratio
*R^2^*	-	coefficient of determination
*Sa*	µm	arithmetical mean height
*Sk*	µm	core height
*Sku*	-	kurtosis
*Ssk*	-	skewness
*Sp*	µm	maximum peak height
*Sq*	µm	root mean square height
*Sv*	µm	maximum pit height
*Sz*	µm	maximum height
*Vmc*	µm³/µm²	
*Vmp*	µm³/µm²	
*Vv*	µm³/µm²	
*Vvc*	µm³/µm²	
*Vvv*	µm³/µm²	
*σ_YS_*	MPa	yield strength
*σ_UTS_*	MPa	ultimate tensile strength
*υ*	-	Poisson’s ratio
*σ_a_*	MPa	maximum nominal stress amplitude
*σ_m_*	MPa	nominal normal mean stress
*τ_a_*	MPa	nominal shear stress amplitude
*τ_m_*	MPa	nominal shear mean stress
